# Cortical asymmetry in autosomal dominant Alzheimer’s disease progression

**DOI:** 10.1093/braincomms/fcaf488

**Published:** 2025-12-19

**Authors:** Agnès Pérez-Millan, Neus Falgàs, Beatriz Bosch, Sergi Borrego-Écija, Anna Antonell, Guadalupe Fernández-Villullas, Diana Esteller-Gauxax, Adrià Tort-Merino, Núria Bargalló, Mircea Balasa, Albert Lladó, David Aguillon, Patricio Chrem, Gregory S Day, Emma Devenney, Edward D Huey, Takeshi Ikeuchi, Mathias Jucker, Kensaku Kasuga, Jonathan Vöglein, Jee Hoon Roh, Paolo Vitali, Ana Luisa Sosa Ortiz, Jorge J Llibre-Guerra, Brian A Gordon, Eric McDade, Randall J Bateman, Raquel Sánchez-Valle, The Dominantly Inherited Alzheimer Network (DIAN), Randall Bateman, Alisha J Daniels, Laura Courtney, Angela Ziegemeier, Karina Skrbec, Cortaiga Hellm, Mariana Martin, Ellen Ziegemeier, Jamie Bartzel, Eric McDade, Jorge J Llibre-Guerra, Charlene Supnet-Bell, Chengie Xiong, Xiong Xu, Ruijin Lu, Guoqiao Wang, Yan Li, Yuzheng Nie, Emily Gremminger, Richard J Perrin, Erin Franklin, Laura Ibanez, Gina Jerome, Jennifer Stauber, Bryce Baker, Matthew Minton, Carlos Cruchaga, Alison M Goate, Alan E Renton, Danielle M Picarello, Brian Fulton-Howard, Tammie L S Benzinger, Brian A Gordon, Jessica Banks, Russ Hornbeck, Allison Chen, Charles Chen, Shaney Flores, Manu Goyal, Diana Hobbs, Nelly Joseph-Mathurin, Kelley Jackson, Sarah Keefe, Deborah Koudelis, Parinaz Massoumzadeh, Austin McCullough, Nicole McKay, Joyce Nicklaus, Christine Pulizos, Qing Wang, Edita Sabaredzovic, Jalen Scott, Ashlee Simmons, Jacqueline Rizzo, Andrei Vlassenko, Yong Wang, Jason Hassenstab, Jennifer Smith, Sarah Stout, Andrew J Aschenbrenner, Celeste M Karch, Jacob Marsh, John C Morris, David M Holtzman, Nicolas R Barthélemy, Jinbin Xu, Sarah B Berman, Snezana Ikonomovic, Gregory S Day, Neill R Graff-Radford, Martin Farlow, Jasmeer P Chhatwal, Courtney Maa, Takeshi Ikeuchi, Kensaku Kasuga, Takanobu Ishiguro, Kenji Ishii, Michio Senda, Yoshiki Niimi, Edward D Huey, Stephen Salloway, Emma Devenney, Peter R Schofield, William S Brooks, Jacob A Bechara, Ralph Martins, Nick C Fox, David M Cash, Natalie S Ryan, Mathias Jucker, Christoph Laske, Reda Timofejavaite, Elke Kuder-Buletta, Susanne Graber-Sultan, Christian la Fougère, Gerald Reischl, Ulrike Obermueller, Johannes Levin, Yvonne Rödenbeck, Jonathan Vöglein, Jae-Hong Lee, Jee Hoon Roh, Paolo Vitali, Ricardo F Allegri, Patricio Chrem Mendez, Ezequiel Surace, Silvia Vazquez, David Aguillon, Yudy Milena Leon, Laura Ramirez, Laura Serna, Ana Baena, Yamile Bocanegra, Allan I Levey, Erik C B Johnson, Nicholas T Seyfried, John Ringman, Anne M Fagan, Hiroshi Mori, Colin Masters, James M Noble, Raquel Sanchez-Valle, Francisco Lopera

**Affiliations:** Alzheimer’s Disease and Other Cognitive Disorders Group, Service of Neurology, Hospital Clínic de Barcelona. Fundació Recerca Clínic Barcelona-IDIBAPS, Barcelona 08036, Spain; Faculty of Computer Science, Multimedia and Telecommunications, Universitat Oberta de Catalunya, Barcelona 08016, Spain; Centro de Investigación Biomédica en Red en Enfermedades Neurodegenerativas (CIBERNED), Madrid 28029, Spain; Alzheimer’s Disease and Other Cognitive Disorders Group, Service of Neurology, Hospital Clínic de Barcelona. Fundació Recerca Clínic Barcelona-IDIBAPS, Barcelona 08036, Spain; Centro de Investigación Biomédica en Red en Enfermedades Neurodegenerativas (CIBERNED), Madrid 28029, Spain; Alzheimer’s Disease and Other Cognitive Disorders Group, Service of Neurology, Hospital Clínic de Barcelona. Fundació Recerca Clínic Barcelona-IDIBAPS, Barcelona 08036, Spain; Centro de Investigación Biomédica en Red en Enfermedades Neurodegenerativas (CIBERNED), Madrid 28029, Spain; Alzheimer’s Disease and Other Cognitive Disorders Group, Service of Neurology, Hospital Clínic de Barcelona. Fundació Recerca Clínic Barcelona-IDIBAPS, Barcelona 08036, Spain; Centro de Investigación Biomédica en Red en Enfermedades Neurodegenerativas (CIBERNED), Madrid 28029, Spain; Alzheimer’s Disease and Other Cognitive Disorders Group, Service of Neurology, Hospital Clínic de Barcelona. Fundació Recerca Clínic Barcelona-IDIBAPS, Barcelona 08036, Spain; Centro de Investigación Biomédica en Red en Enfermedades Neurodegenerativas (CIBERNED), Madrid 28029, Spain; Alzheimer’s Disease and Other Cognitive Disorders Group, Service of Neurology, Hospital Clínic de Barcelona. Fundació Recerca Clínic Barcelona-IDIBAPS, Barcelona 08036, Spain; Centro de Investigación Biomédica en Red en Enfermedades Neurodegenerativas (CIBERNED), Madrid 28029, Spain; Alzheimer’s Disease and Other Cognitive Disorders Group, Service of Neurology, Hospital Clínic de Barcelona. Fundació Recerca Clínic Barcelona-IDIBAPS, Barcelona 08036, Spain; Departament de Medicina, Facultat de Medicina i Ciències de la Salut, Universitat de Barcelona, Barcelona 08036, Spain; Alzheimer’s Disease and Other Cognitive Disorders Group, Service of Neurology, Hospital Clínic de Barcelona. Fundació Recerca Clínic Barcelona-IDIBAPS, Barcelona 08036, Spain; Centro de Investigación Biomédica en Red en Enfermedades Neurodegenerativas (CIBERNED), Madrid 28029, Spain; Image Diagnostic Centre, Hospital Clínic de Barcelona, Barcelona 08036, Spain; CIBER de Salud Mental, Instituto de Salud Carlos III, Magnetic Resonance Image Core Facility, IDIBAPS, Barcelona 08036, Spain; Alzheimer’s Disease and Other Cognitive Disorders Group, Service of Neurology, Hospital Clínic de Barcelona. Fundació Recerca Clínic Barcelona-IDIBAPS, Barcelona 08036, Spain; Centro de Investigación Biomédica en Red en Enfermedades Neurodegenerativas (CIBERNED), Madrid 28029, Spain; Alzheimer’s Disease and Other Cognitive Disorders Group, Service of Neurology, Hospital Clínic de Barcelona. Fundació Recerca Clínic Barcelona-IDIBAPS, Barcelona 08036, Spain; Centro de Investigación Biomédica en Red en Enfermedades Neurodegenerativas (CIBERNED), Madrid 28029, Spain; Departament de Medicina, Facultat de Medicina i Ciències de la Salut, Universitat de Barcelona, Barcelona 08036, Spain; Grupo de Neurociencias de Antioquia (GNA), Facultad de Medicina, Universidad de Antioquia, Medellín 050010, Colombia; Institute of Neurological Research, FLENI, Buenos Aires C1428AQK, Argentina; Department of Neurology, Mayo Clinic in Florida, Jacksonville, FL 32224, USA; Neuroscience Research Australia, Sydney 2031, Australia; Department of Psychiatry and Human Behavior, Alpert Medical School, Brown University, Providence 02912, USA; Niigata University, Niigata 8050, Japan; German Center for Neurodegenerative Diseases (DZNE), Tübingen 72076, Germany; Hertie Institute for Clinical Brain Research, University of Tübingen, Tübingen 72076, Germany; Brain Research Institute, Niigata University, Niigata 8050, Japan; Department of Neurology, LMU University Hospital, LMU Munich, Munich 81377, Germany; German Center for Neurodegenerative Diseases (DZNE), Munich 81377, Germany; Departments of Physiology and Neurology, Korea University Anam Hospital, Korea University College of Medicine, Seoul 02841, Korea; Translational Neuroimaging Laboratory, Department of Neurology and Neurosurgery, Psychiatry and Pharmacology and Therapeutics, McGill University Research Centre for Studies in Aging, Montreal Neurological Institute-Hospital, Douglas Research Institute, McGill University, Montreal, Canada H3A 2B4; Dementias Laboratory, National Institute of Neurology and Neurosurgery, Mexico City, DF 14269, Mexico; Department of Neurology, Washington University School of Medicine, St. Louis, MO 63110, USA; Department of Neurology, Washington University School of Medicine, St. Louis, MO 63110, USA; Department of Neurology, Washington University School of Medicine, St. Louis, MO 63110, USA; Department of Neurology, Washington University School of Medicine, St. Louis, MO 63110, USA; Alzheimer’s Disease and Other Cognitive Disorders Group, Service of Neurology, Hospital Clínic de Barcelona. Fundació Recerca Clínic Barcelona-IDIBAPS, Barcelona 08036, Spain; Centro de Investigación Biomédica en Red en Enfermedades Neurodegenerativas (CIBERNED), Madrid 28029, Spain; Departament de Medicina, Facultat de Medicina i Ciències de la Salut, Universitat de Barcelona, Barcelona 08036, Spain

**Keywords:** brain, Alzheimer’s disease, autosomal dominant Alzheimer’s disease, magnetic resonance imaging, APOE

## Abstract

The cortical asymmetry index evaluates the cortical thickness asymmetry between hemispheres. We investigated cortical asymmetry index in asymptomatic and symptomatic mutation carriers of autosomal dominant Alzheimer’s disease to explore the brain asymmetry within the Alzheimer’s disease continuum. Sixty baseline T1-weighted MRI scans were obtained from the Clinic Barcelona cohort. Baseline and longitudinal MRI data from 564 participants within the dominantly inherited Alzheimer network observational study were used as an independent, confirmatory cohort. Cerebrospinal fluid and plasma neurofilament light chain levels were included when available. Cortical thickness was calculated using Freesurfer and cortical asymmetry index was calculated via an open-source pipeline. Cross-sectional analyses examined cortical asymmetry index differences based on clinical classification and *APOE* ε*4* status, adjusting for age, sex and estimated years from onset, while correlations were assessed with age, estimated years from onset, mini-mental state examination scores, and neurofilament light. Longitudinal cortical asymmetry index evolution was modelled using generalized additive models in the dominantly inherited Alzheimer network observational study cohort, incorporating age, sex, and the interaction between group and estimated years from onset. The cortical asymmetry index successfully distinguished asymptomatic mutation carrier and symptomatic mutation carriers from healthy controls in the Clinic Barcelona cohort and symptomatic mutation carriers from controls in dominantly inherited Alzheimer network observational study. Higher cortical asymmetry index in mutation carriers (asymptomatic mutation carrier and symptomatic mutation carriers combined) and in symptomatic mutation carriers were associated with higher plasma neurofilament light levels, a closer proximity to symptom onset, and lower mini-mental state examination in the Clinic Barcelona cohort. In the dominantly inherited Alzheimer network observational study cohort, mutation carriers exhibited increased cortical asymmetry index compared to controls and correlated with elevated neurofilament light (plasma and Cerebrospinal fluid), lower mini-mental state examination, and a closer proximity to symptom onset. *APOE3/3* carriers showed greater asymmetry than other *APOE* genotypes and significant cortical asymmetry index differences between asymptomatic mutation carrier and symptomatic mutation carriers. Longitudinally, cortical asymmetry index increased over time significantly in symptomatic mutation carriers. These findings underscore brain asymmetry as a potential biomarker for early Alzheimer’s disease progression in autosomal dominant Alzheimer’s disease, with implications for detection and monitoring tracking disease-related neuroanatomical changes.

## Teaser

Pérez-Millan et al. report that cortical asymmetry distinguishes mutation carriers of autosomal dominant Alzheimer’s disease from healthy controls and differentiates asymptomatic carriers. Higher asymmetry was correlated with neurofilament light chain, cognitive decline, and proximity to symptom onset. Longitudinal analyses showed progression in symptomatic carriers, supporting asymmetry as an early biomarker.

## Graphical Abstract

**Graphical Abstract:**
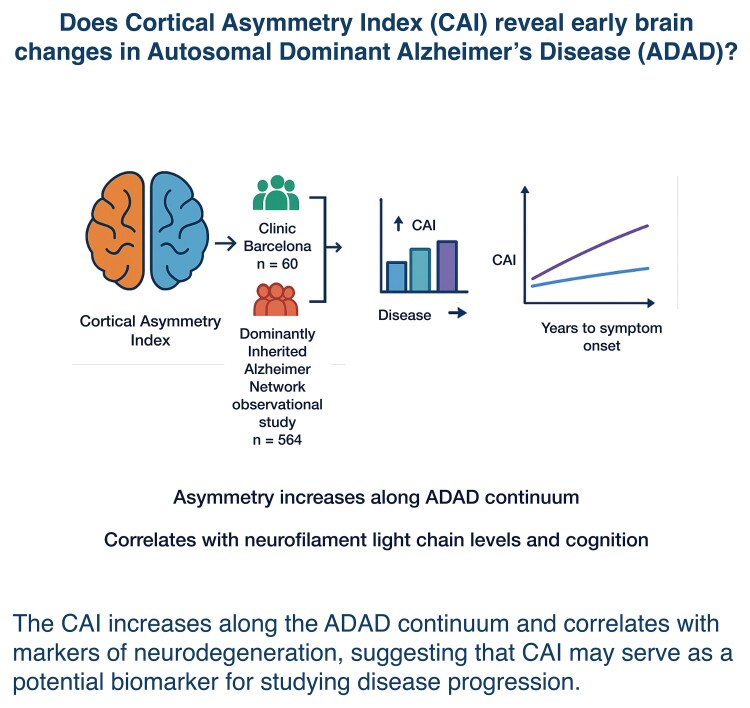

## Introduction

Alzheimer’s disease (AD) leads to progressive cortical atrophy, particularly in the hippocampal and temporoparietal cortices, which have been typically described at visual inspection as symmetrical between the left and right hemispheres. However, subtle brain asymmetric changes can be identified using advanced neuroimaging techniques. In this regard, several recent studies investigating brain asymmetries have described the presence of asymmetric patterns in individuals with Alzheimer’s disease.^1-4^ For instance, Lubben *et al*. and Roe *et al*. showed that the cortical thinning of AD participants measured by cortical thickness is more pronounced in the left hemisphere.^3,4^ This asymmetry in AD is evident not only compared to healthy controls (CTR) but also in individuals with mild cognitive impairment (MCI), which precedes AD dementia.^5-8^ Therefore, developing neuroimaging markers of brain asymmetry could be helpful for early disease detection.

While differences between structural measures from the left and right hemispheres can be detected in structural magnetic resonance imaging (MRI), no established method exists to calculate brain asymmetry. In 2025, we proposed the cortical asymmetry index (CAI),^9^ a measure designed to assess brain asymmetry using cortical thickness probability distributions from each hemisphere. This index estimates individual asymmetry based on entropy calculations. In this previous work, we tested CAI in a cohort of individuals with Alzheimer’s disease and frontotemporal dementia (FTD), which confirmed the capacity of this marker to estimate brain asymmetry and discriminate between AD and FTD.

Despite the recent advancements, most research in the field has focused on sporadic AD, and there is a lack of studies investigating brain asymmetry in individuals with Autosomal Dominant Alzheimer’s Disease (ADAD). The ADAD population provides the opportunity to evaluate biomarkers from the pre-symptomatic stage until the phase of dementia. Studying these stages, including pre-clinical AD, would be the key to understanding brain asymmetry phenomena and how it progresses along the AD continuum; this could be particularly relevant considering prior reports suggesting that brain asymmetry may precede the onset of the symptoms,^10^ suggesting this marker could have value to monitor disease progression. *APOE* ε*4+* status has been proposed as a factor influencing not only the earlier age of onset of AD but also greater memory impairment,^11^ and increased tau deposition and atrophy.^12^ Despite its relevance in many aspects of brain changes within the AD spectrum, whether *APOE* status shows different asymmetry patterns has never been studied.

Cerebrospinal fluid (CSF), blood, and neuroimaging biomarkers can be analysed to detect or monitor the pathological changes *in vivo* caused by neurodegeneration.^13,14^ Neurofilament light chain (NfL) is a marker of axonal damage and neurodegeneration that is elevated in various neurodegenerative disorders, including AD.^15-17^ In AD, elevated levels of NfL have been associated with greater brain atrophy, in particular in brain regions affected by AD.^18-20^ However, the association between NfL levels and brain asymmetry is still unclear.

In summary, in this study, we aim to investigate brain asymmetry using the CAI in ADAD (including a uni-centric cohort and a multi-centric replication cohort), to explore its potential as an early biomarker for AD, also considering the *APOE* status. In addition, we aim to assess brain asymmetry longitudinally and evaluate its correlation with global cognition (MMSE) and neurodegeneration markers (NfL) to determine if CAI can track AD progression.

## Material and methods

### Participants

#### Clinic Barcelona cohort

The study includes 60 participants recruited from the ADAD cohort at Hospital Clínic de Barcelona (the Clinic Barcelona cohort), including individuals carrying *PSEN1* or *APP* gene mutations. Baseline T1-weighted MRIs were included for symptomatic mutation carriers (SMC) (CDR ≥ 0.5), asymptomatic mutation carriers (AMC) (CDR = 0), and healthy controls (CTR) (CDR = 0). Available CSF and plasma NfL levels were included. Participants with a history of stroke, traumatic brain injury, major psychiatric disorder, or alcohol abuse were excluded.

This study was performed according to the international consensus for research with human subjects (the updated version of Helsinki’s Statement, Fortaleza, 2013) and Spanish regulations. The Hospital Clínic de Barcelona Ethics Committee approved the study (HCB/2020/1410), and all the participants signed the informed consent.

#### Dominantly inherited Alzheimer network observational study cohort

All data came from participants enrolled in dominantly inherited Alzheimer network observational study (DIAN), a worldwide, multi-modal study of ADAD mutation carriers and non-carrier family members^21^ from data freeze 17 (2023), who serve as a replication and longitudinal cohort (called DIAN-OBS cohort). The appropriate Institutional Review Boards and research ethics committee reviewed and approved the DIAN study for each participating site. Informed consent was obtained from all participants. Participant enrolment was carried out according to pre-specified inclusion and exclusion criteria described previously.^22^

Participants in DIAN-OBS come from families known to carry a pathological mutation in *PSEN1*, *PSEN2*, or *APP* genes. We included data from T1-weighted MRI images of 564 participants, some of whom have longitudinal data. We include SMC (CDR ≥ 0.5), AMC (CDR = 0), and CTR (CDR = 0). In the DIAN-OBS cohort, SMC participants were subdivided into mild cognitive impairment (SMC-MCI, CDR = 0.5) and Alzheimer’s disease (SMC-AD, CDR ≥ 1). Available CSF and plasma NfL levels were included.

#### Estimated years from symptom onset

Estimated years from symptom onset (EYO) were calculated for individual participants by subtracting the age at which their affected parent first developed symptoms from the participant’s age at their visit since age at symptomatic onset is relatively consistent within ADAD families.^23,24^

### Biochemical markers and *APOE* genotype

#### Clinic Barcelona cohort

Both CSF and plasma NfL were measured at the AD and other cognitive disorders group laboratory in Barcelona, Spain, using the single-molecule array Quanterix Neurology 4-Plex A, as described in Pérez-Millan *et al*.^9^

#### DIAN-OBS cohort

The central biomarker core at Washington University measured the CSF and plasma NfL levels. The CSF and plasma NfL level was performed following a protocol consistent with Alzheimer’s disease neuroimaging initiative (ADNI).^25,26^

The DIAN Genetics Core at Washington University performed the *APOE* genotyping using PCR-based amplification of the appropriate exon followed by Sanger sequencing.^22,25^ DIAN determined *APOE* genotype using an ABI pre-designed real-time TaqMan assay. *APOE* carriers’ status was analysed according to the presence or absence of at least one APOE ε4 allele.

### MRI data acquisition and pre-processing

#### Clinic Barcelona cohort

All participants were scanned in a 3T scanner using the 3D T1-weighted MPRAGE sequence described in Pérez-Millan *et al*.^27^

We used the processing stream available in FreeSurfer version 6.0 (http://surfer.nmr.mgh.harvard.edu.sire.ub.edu/) to perform cortical reconstruction of the T1-weighted acquisitions following the same procedure as previous papers of the group Pérez-Millan *et al*. and described in FreeSurfer papers previously.^9,28-30^ We obtain mean cortical thickness measures for 68 cortical parcellations (34 per hemisphere) derived from the Desikan–Killiany atlas.^31^ The segmentations were visually inspected and corrected if needed.

#### DIAN-OBS cohort

Structural MRI acquisition was performed using the ADNI protocol.^32,33^ Each participant received an accelerated 3D sagittal T1-weighted MPRAGE on a 3T scanner. Before analysis, all images had passed quality control assessments to ensure acquisition conformity, and the ADNI imaging core screened them for artefacts and protocol compliance.

The DIAN Imaging Core at Washington University processed MRI images using FreeSurfer 5.3^24,34^ to perform cortical reconstruction of the T1-weighted acquisitions needed to estimate the cortical thickness, similar to the Clinic Barcelona cohort.

#### Cortical asymmetry index

We obtained the CAI by implementing a measure derived from the Jensen-Shannon distance, a methodology based on a metric derived from information theory from a previous group paper^9^ and available code at https://github.com/Agnes2/CAI. We obtained the CAI using the distribution of cortical thickness measures of each brain hemisphere (previously obtained with the software FreeSurfer) for each cohort independently. CAI quantifies brain structural asymmetry at the individual level and is an a-dimensional measure with higher values indicating a more asymmetric brain. The CAI was calculated in Python version 3.10.6 (https://www.python.org).

### Statistical analysis

We compared the demographic and clinical data among groups using the Kruskal–Wallis test for continuous variables, and the Fisher test for discrete variables in the Clinic Barcelona cohort, and the ANOVA test for continuous variables, and the Fisher test for discrete variables in the DIAN-OBS cohort. Continuous variables were expressed as mean ± standard deviation (SD).

Using a permutation test, we first compared the CAI between mutation carriers versus CTR at baseline, adding age, sex, and EYO as covariates. Then, all diagnosis groups, including CTR, AMC, and SMC participants for both cohorts, were analysed at baseline. We used the permutation test, correcting for age, sex, and EYO in the Barcelona clinic cohort, and ANCOVA, adding age, sex, and EYO as covariates in the DIAN-OBS cohort. Finally, in the DIAN-OBS cohort, we examined the genes (*PSEN1* and *APP*) separately. Receiver operating characteristic curves were analysed to assess the diagnostic accuracy of CAI compared to hippocampus volume and NfL in both cohorts.

In the DIAN-OBS cohort baseline, we studied *APOE ɛ4* differences in mutation carriers, adjusting by age, sex, EYO, and CDR using a permutation test. First, we examined the differences between the different *APOE* genotypes in all the mutation carriers. Then, we studied the differences between AMC and SMC for each *APOE* genotype. Finally, we examined the *APOE* genotypes for the genes (*PSEN1* and *APP*) separately.

We evaluated the association between CAI and age, EYO, MMSE, and NfL (CSF and plasma) in both cohorts at baseline. We used Spearman’s rank correlation coefficient due to the limited samples in the Clinic Barcelona cohort and Pearson’s in the DIAN-OBS cohort.

Finally, we performed longitudinal analyses in the DIAN-OBS cohort to study progression differences using generalized additive model (GAM) with baseline plus six follow-ups (when available) according to EYO; we added as fixed effects group, EYO, the interaction between them, age, and sex.

We implemented the statistical analyses in R version 4.2.1. The significance level was set in all the analyses at a *P*-value < 0.05, correcting the pairwise differences using Benjamini–Hochberg correction.

## Results

### Descriptive analysis

Of the 60 participants (68% female) in the Clinic Barcelona cohort, 19 were SMC, 22 AMC, and 19 CTR (Table 1). In the DIAN-OBS cohort at baseline (Table 2), we include 115 SMC, 234 AMC, and 215 CTR (58% female). Then, in the DIAN-OBS cohort, SMC participants were subdivided into SMC-MCI (*N* = 73, CDR = 0.5) and SMC-AD (*N* = 42, CDR ≥ 1).

**Table 1:** Demographics of participants by genetic status for clinic Barcelona cohort

	CTR	AMC	SMC	*P*-values
N MRI	19	22	19	
Sex, men/women	7/12	4/18	8/11	0.24
Age at MRI, years (SD)	33.7 (10.9)	37.0 (8.9)	50.5 (10.0)	**<0.0001**
EYO, years (SD)	−6.4 (7.9)	−9.4 (10.7)	2.4 (8.1)	**<0.001**
APOE, carrier/no carrier	5/13	2/17	2/16	0.43
MMSE, (SD)	29.3 (1.2)	29.3 (1.0)	20.1 (5.6)	**<0.0001**
CSF NfL, pg/ml (SD)	263.3 (99.4)	283.6 (67.2)	1073.5 (294.9)	**<0.0001**
Plasma Nfl, pg/ml (SD)	5.7 (2.4)	7.8 (4.3)	17.3 (7.5)	**<0.0001**

We highlighted the significant *P*-values in bold.

CTR, healthy controls; AMC, asymptomatic mutation carriers; SMC, symptomatic mutation carriers; CSF, cerebrospinal fluid; NfL, neurofilament-light chain; EYO, estimated year to onset; MMSE, mini-mental state examination.

**Table 2:** Demographics of participants by genetic status for DIAN-OBS cohort

	CTR	AMC	SMC	*P*-values
*N* MRI	215	234	115	
Sex at MRI, men/women	90/125	100/134	49/66	0.98
Age at MRI, years (SD)	37.2 (11.0)	34.2 (9.3)	45.2 (9.7)	**<0.0001**
EYO, years (SD)	−10.4 (11.8)	−14.1 (8.6)	0.5 (8.0)	**<0.0001**
APOE, carrier/no carrier	67/148	69/165	31/84	0.73
MMSE (SD)	29.1 (1.3)	29.1 (1.2)	22.9 (6.5)	**<0.0001**
CSF NfL, pg/ml (SD)	250.1 (153.4)	267.4 (169.1)	945.6 (568.7)	**<0.0001**
Plasma NfL, pg/ml (SD)	6.3 (4.6)	6.0 (2.8)	15.1 (10.0)	**<0.0001**

We highlighted the significant *P*-values in bold.

CTR, healthy controls; AMC, asymptomatic mutation carriers; SMC, symptomatic mutation carriers; CSF, cerebrospinal fluid; NfL, neurofilament-light chain; EYO, estimated year to onset; MMSE, mini-mental state examination, *N* MRI, number of participants who completed the T1-weighted MRI scan.

As expected, significant differences between CTR, AMC, and SMC participants were found for age, EYO, MMSE, and NfL in both cohorts (Tables 1 and 2). There were no significant group differences in sex, although there were more women than men in all the groups in both cohorts. MMSE scores in both cohorts are similar. In the DIAN-OBS cohort at baseline (Table 2), there were no significant differences in the distribution of *APOE* carriers between groups.

In the DIAN-OBS cohort, AMC and SMC patients were younger than in the Barcelona Clinic cohort (*P*-value < 0.001 and *P*-value < 0.05, respectively). Additionally, the AMC participants in the DIAN-OBS cohort exhibit a higher negative EYO (*P*-value < 0.01), indicating that they are further away from the expected age of conversion than the Clinic Barcelona cohort.

### CAI by clinical status in Clinic Barcelona and DIAN-OBS cohorts

#### Clinic Barcelona cohort

**Figure 1:**
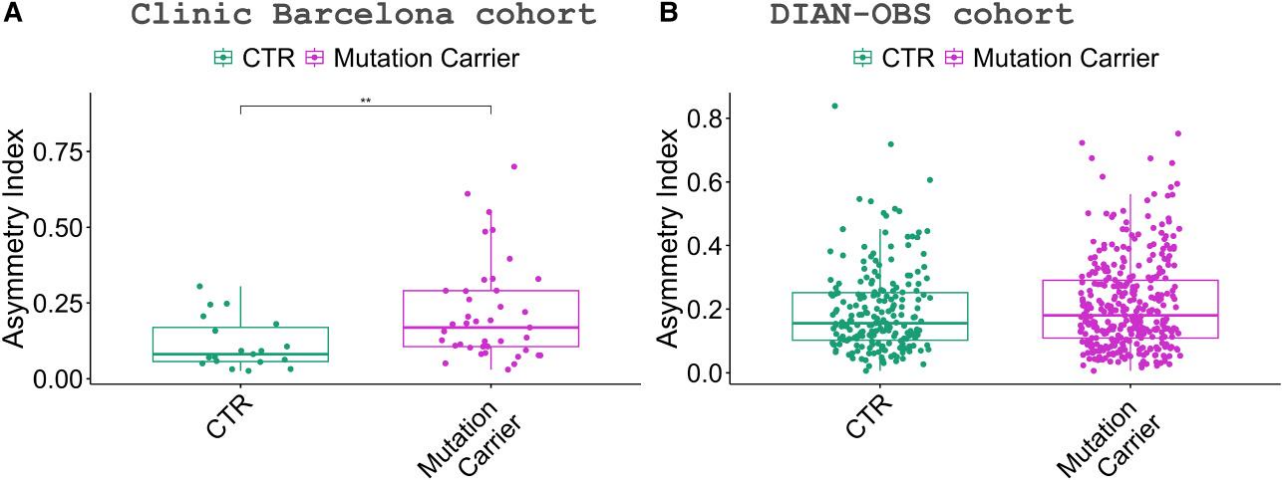
**Asymmetric index showing significant differences between healthy controls (CTR) and mutation carriers (AMCs and SMCs), analysed with a permutation test.** (**A**) Clinic Barcelona cohort results with CTR (*N* = 19) and mutation carriers (*N* = 41) (**B**) DIAN-OBS cohort results with CTR (*N* = 215) and mutation carriers (*N* = 349). Each data point represents the asymmetric index of one individual participant. Symbols indicate significance levels **P-value < 0.01.

**Figure 2:**
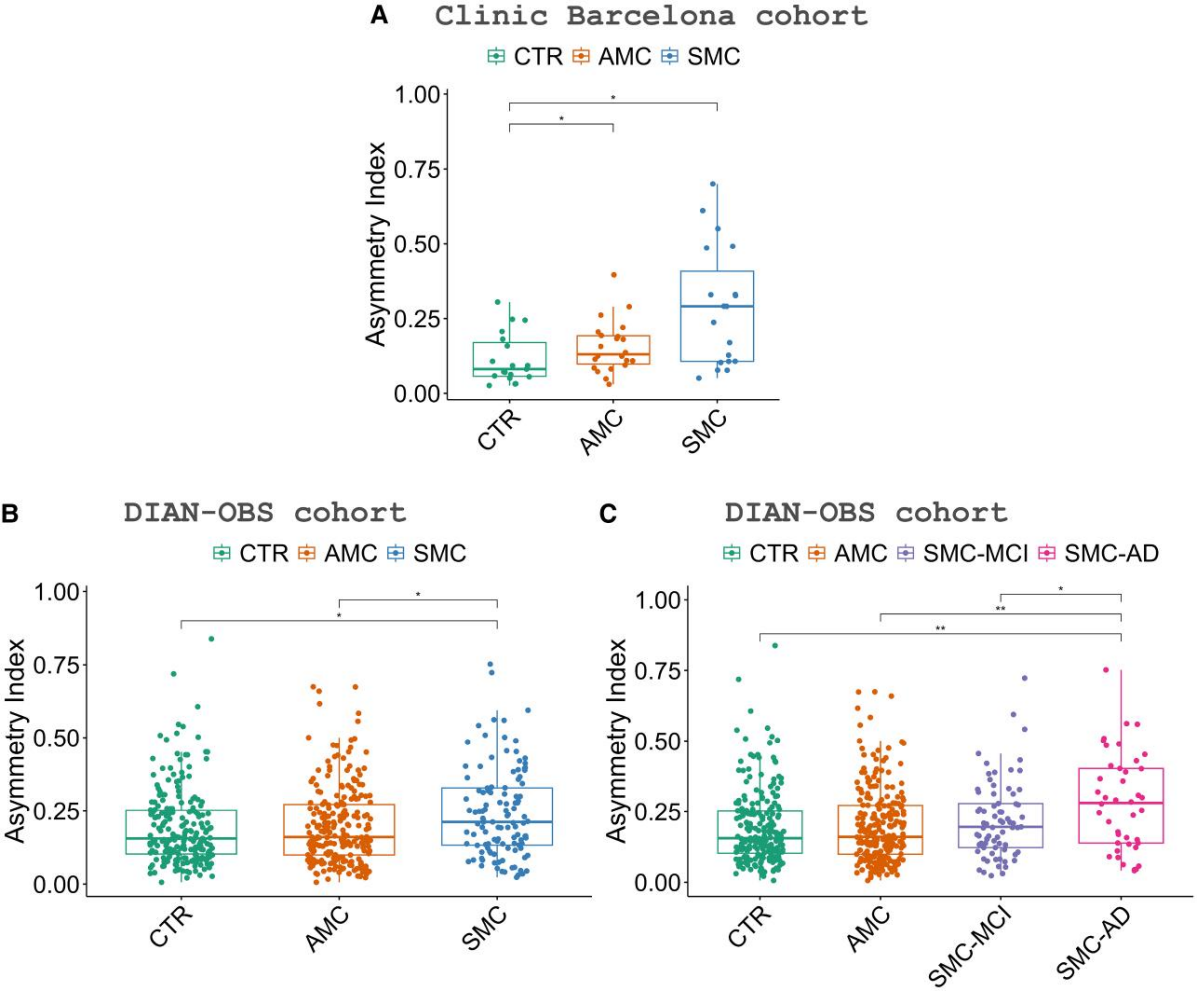
**Asymmetric index showing significant differences between groups.** (**A**) Results in the Clinic Barcelona cohort estimated with a permutation test with healthy controls (CTR) (*N* = 19), AMC (*N* = 22), and SMC (*N* = 19). (**B**) and (**C**) Results in the DIAN-OBS cohort estimated with an ANCOVA test with CTR (*N* = 215), AMC (*N* = 234), SMC (*N* = 115), SMC-MCI (*N* = 73), and SMC-AD (*N* = 42). Each data point represents the asymmetric index of one individual participant. Symbols indicate significance levels: *P -value < 0.05 and **P -value < 0.01.

In the Clinic Barcelona cohort, we found that mutation carriers presented higher CAI values (more asymmetric brain) than CTR (*P*-value < 0.05) (Fig. 1). Both SMC and AMC presented higher CAI than CTR (*P*-value < 0.05) (Fig. 2). We found lower CAI in CTR than AMC and SMC, indicating an asymmetric brain structure due to Alzheimer’s disease. CAI could be used to differentiate SMC from CTR, with an area under the curve (AUC) of 0.8 (AUCs of 0.8 for hippocampus volume and 1.0 for NfL) (Fig. 1; Supplementary material). To differentiate AMC from SMC, AUC 0.7 for CAI (AUCs 0.8 hippocampus and 1.0 NfL). Finally, to discriminate AMC from CTR, CAI AUC 0.7 (AUCs 0.5 hippocampus and 0.6 NfL).

#### DIAN-OBS cohort

The DIAN-OBS cohort (Fig. 2) showed significant differences between SMC and CTR (*P*-value < 0.05), replicating prior findings, but not between AMC and CTR. Additionally, it reveals significant differences between AMC and SMC (*P*-value < 0.05). CAI could be used to differentiate SMC from CTR, with an AUC of 0.6 (AUCs of 0.8 for hippocampus volume and 0.9 for NfL) (Fig. 1; Supplementary material). To differentiate AMC from SMC, AUC 0.6 for CAI (AUCs 0.8 hippocampus and 0.9 NfL). Finally, to discriminate AMC from CTR, CAI AUC 0.5 (AUCs 0.5 hippocampus and 0.5 NfL). Finally, we subdivided the SMC participants according to their CDR, SMC-MCI (CDR = 0) and SMC-AD (CDR ≥ 1). We found that SMC-AD presented higher CAI values compared to all the other diagnostic groups, showing significant differences with SMC-MCI (*P*-value < 0.05) and CTR and AMC (*P*-value < 0.05) (Fig. 2). Finally, we examined the differences between the clinical groups for the individual genes *PSEN1* and *APP*. For *PSEN1*, we found significant differences between CTR and AMC versus SMC-AD (*P*-value < 0.05) (Fig. 2; Supplementary material). For *APP*, we found significant differences between CTR and SMC (*P*-value < 0.05), as well as between CTR and SMC-AD (*P*-value < 0.05) (Fig. 2; Supplementary material).

### CAI by *APOE* genotype in DIAN-OBS cohort

**Figure 3:**
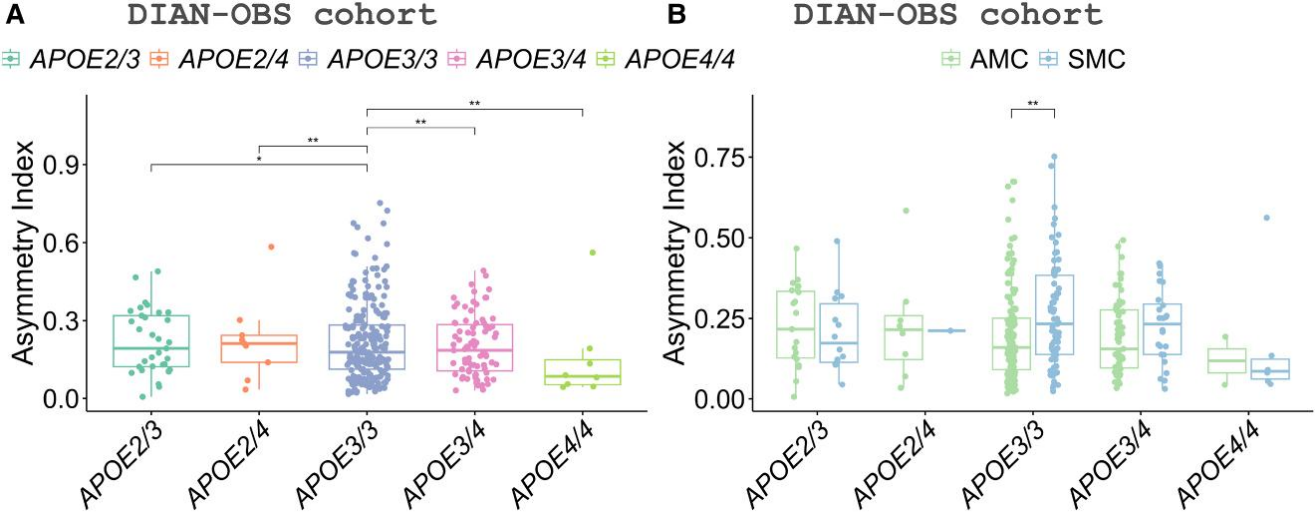
**Asymmetric index showing significant differences between APOE genotypes in the DIAN-OBS cohort analysed with a permutation test.** Groups include AMC (*N* = 233) and SMC (*N* = 114). (**A**) Differences between APOE genotypes for mutation carriers AMC and SMC. (**B**) Differences between AMC and SMC for the different APOE genotypes. Each data point represents the asymmetric index of one individual participant. Symbols indicate significance levels: *P -value < 0.05 and **P -value < 0.01.

In the DIAN-OBS cohort, we first studied the differences between APOE genotypes for the mutation carriers. We found that *APOE3/3* presented higher CAI values compared to the other *APOE* genotypes (Fig. 3). When analysing differences between clinical groups, *APOE3/3* was the only *APOE* genotype showing higher asymmetry in SMC compared to AMC (*P*-value < 0.01) (Fig. 3). The CTR groups did not present significant differences for any *APOE* genotype. Finally, we examined the differences between the clinical groups for the individual genes *PSEN1* and *APP*. For both genes, we found that *APOE3/3* presented higher CAI values compared to the other *APOE* genotypes (*P*-value < 0.05) (Fig. 3; Supplementary material). Additionally, in both cases, *APOE3/3* was the only *APOE* genotype to show higher asymmetry in SMC compared to AMC (*P*-value < 0.05).

### Association between CAI and NfL, MMSE and EYO in clinic Barcelona and DIAN-OBS cohorts

#### Clinic Barcelona cohort

In the Clinic Barcelona cohort for mutation carriers and SMC showed significant correlation between CAI, and plasma-NfL levels (mutation carriers: *r* = 0.47, *P*-value < 0.01; SMC: *r* = 0.72, *P*-value < 0.01), advanced EYO (mutation carriers: *r* = 0.31, *P*-value < 0.05; SMC: *r* = 0.49, *P*-value < 0.05), and lower MMSE (mutation carriers: *r* = −0.32, *P*-value < 0.05; SMC: *r* = −0.55, *P*-value < 0.05). Age did not show any significant correlation with CAI.

#### DIAN-OBS cohort

In the DIAN-OBS cohort, carriers showed higher CAI associated with elevated plasma-NfL (*r* = 0.17, *P*-value < 0.01) and CSF-NfL (*r* = 0.13, *P*-value < 0.5), reduced MMSE (*r* = −0.14, *P*-value < 0.01), and advanced EYO (*r* = 0.15, *P*-value < 0.01). Then, we studied for AMC, SMC, SMC-MCI, and SMC-AD, we found an association for SMC-AD with EYO (*r* = 0.32, *P*-value < 0.05). Age did not show any significant correlation.

### Longitudinal CAI changes in the DIAN-OBS cohort

**Figure 4:**
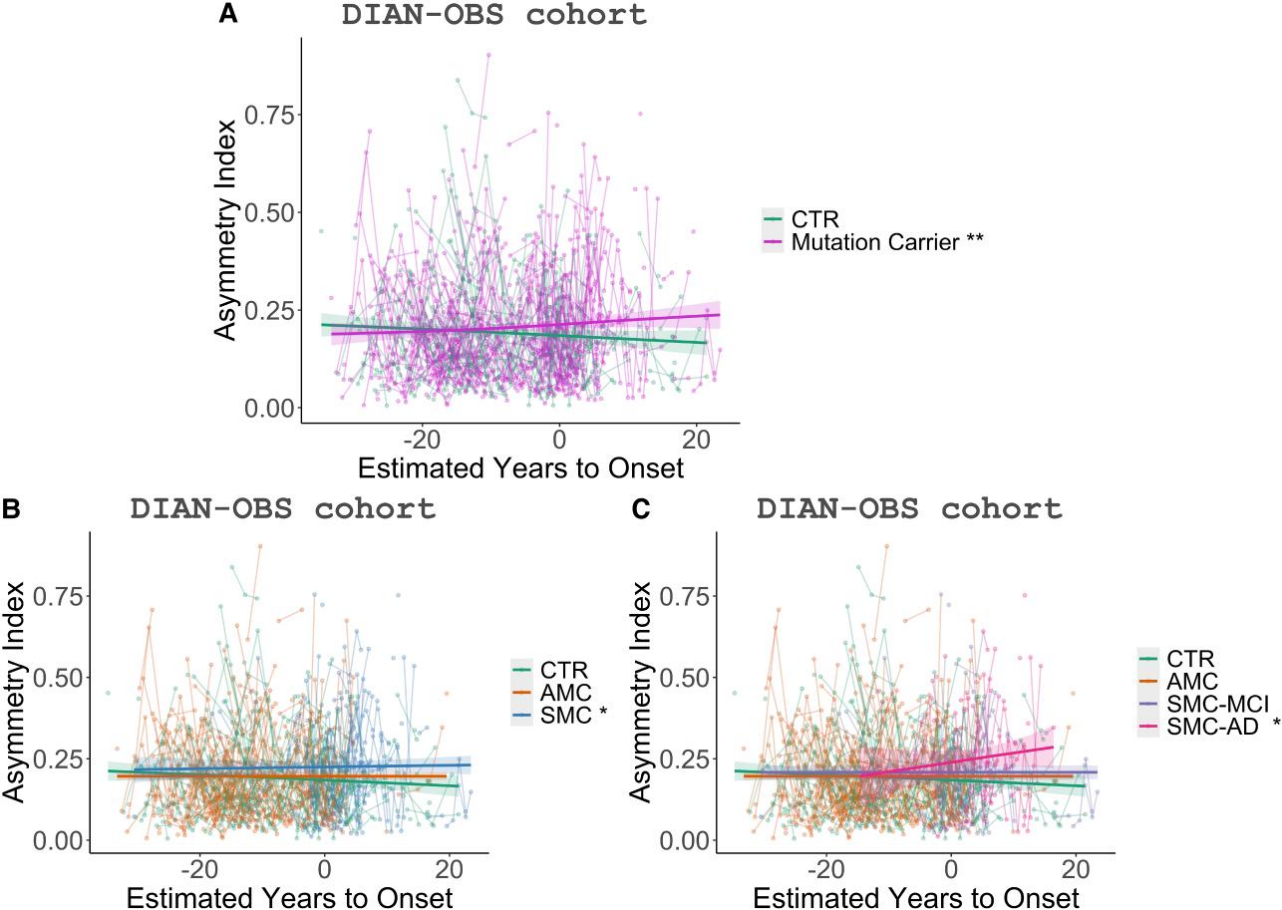
**Trajectories of the asymmetric index according to the parental estimated years to onset in the DIAN-OBS cohort with GAM models.** (**A**) CTR (*N* = 215) and mutation carriers (*N* = 349) trajectories (*t* = 3.06, *P* = 0.0023). (**B**) CTR (*N* = 215), AMC (*N* = 234), and SMC (*N* = 115) trajectories (*t* = 2.13, *P* = 0.033). (**C**) CTR (*N* = 215), AMC (*N* = 234), SMC-MCI (*N* = 73), and SMC-AD (*N* = 42) trajectories (*t* = 2.30, *P* = 0.022). Groups include healthy CTR, AMC, SMC, mild cognitive impairment (SMC-MCI), and Alzheimer’s disease (SMC-AD). Each data point represents the asymmetric index for one individual participant, and the lines connecting the points indicate individual trajectories. Symbols indicate significance levels: *P -value< 0.05 and **P-value < 0.01.

Mutation carriers present a significant increase in CAI (more asymmetry) over time (*P*-value < 0.01) (Fig. 4). When analysing them separately AMC and SMC, we found that the SMC group showed a significant increase in CAI over time (*P*-value < 0.05). In addition, SMC-AD presented a significant CAI increase over time (*P*-value < 0.05) with a higher slope compared to other groups. CTR and AMC showed no significant differences over time (Fig. 4) during the follow-up period.

## Discussion

This study demonstrates the utility of CAI as a tool to evaluate brain asymmetry in ADAD, which increases with disease progression. The findings from the Clinic Barcelona cohort and DIAN-OBS cohort suggest that CAI can be used to differentiate not only between CTR and mutation carriers, but also between those with SMC and AMC individuals. Within SMC, CAI increases with disease progression. *APOE3/3* genotype exhibited greater brain asymmetry than other *APOE* genotypes, even without clinical symptoms. Furthermore, higher CAI values were consistently correlated with elevated NfL levels of both CSF and plasma, which reinforces the hypothesis that brain asymmetry reflects underlying neurodegenerative processes.

This study validates the utility of the CAI as a neuroimaging marker of brain asymmetry by replicating previously published findings in AD and FTD in the Hospital Clínic Barcelona cohort.^9^ By extending its validation to a multi-centric cohort, DIAN-OBS, we reinforce its reliability as an established biomarker rather than a newly introduced measure. Furthermore, our findings confirm that AD exhibits greater asymmetry than previously recognized, not only in its sporadic form, as shown in prior studies, but also in ADAD. Additionally, we can detect brain asymmetry in pre-clinical forms of Alzheimer’s disease.

SMC patients exhibited higher brain asymmetry levels than AMC and CTR, a result replicated in two independent cohorts: Clinic Barcelona and DIAN-OBS. This aligns with previous literature, which indicates that AD patients show greater asymmetry than cognitively healthy individuals.^4,9,10,35,36^ Furthermore, the CAI measured in the Clinic Barcelona cohort differentiated AMC from CTR. To our knowledge, no previous studies have explored brain asymmetry differences between AMC and CTR within an ADAD cohort. Thus, CAI emerges as the first technique capable of measuring cortical brain asymmetry to differentiate AMC from CTR. However, this result has not been replicated in DIAN-OBS cohort. The modest CAI differences between AMC and CTR in the DIAN-OBS cohort may reflect cohort variability and the possibility, as suggested in some studies,^37^ that asymmetry does not uniformly increase with disease progression. We believe the discrepancy between cohorts may be due to significant differences in EYO between the cohorts for AMC individuals. In the Barcelona Clinic cohort, the mean EYO for AMC individuals was −9.4 (10.7) years, whereas in the DIAN cohort, it was −14.1 (8.6) years. Previous studies have shown that EYO can influence brain atrophy and, consequently, brain asymmetry in Alzheimer’s disease,^34,38-40^ as well as in other neurodegenerative diseases such as FTD.^41^

Moreover, CAI further distinguished between SMC-MCI and SMC-AD in the DIAN-OBS cohort, highlighting its potential in identifying those at more advanced stages of Alzheimer’s disease and could be used to track disease progression. Consistent with our findings, other studies have shown that MCI individuals had lower brain asymmetry than Alzheimer’s disease patients in sporadic AD, as measured with various techniques for assessing brain asymmetry.^1,10,35,42^ All these findings support the hypothesis that brain asymmetry, measured through CAI, is a hallmark of early neurodegenerative processes, which can precede the clinical manifestations of dementia. The studies by Long *et al*.^35^ and Wachinger *et al*.^10^ propose that brain asymmetries could serve as an imagining biomarker for the early pre-symptomatic or pre-clinical classification and prediction of AD, based on their findings from the ADNI dataset (https://adni.loni.usc.edu). However, these results remained preliminary and have yet to be conclusively validated. In contrast, our study involving ADAD individuals provides confirmation that brain asymmetry could be a valuable biomarker for tracking disease progression.

The possible influence of *APOE* genotype on brain asymmetry is less clear, although *APOE* effects are known to be regionally specific.^43-47^ Another significant finding of the study is the influence of the *APOE* genotype on brain asymmetry. *APOE3/3* carriers exhibited greater asymmetry in CAI than individuals with other *APOE* genotypes. The APOE ε3 allele is considered the most common and neutral variant in the context of AD, as it has been shown to neither increase nor decrease the risk influence of disease onset.^48-51^ The finding that *APOE3/3* carriers have greater brain asymmetry suggests that genetic pre-disposition plays a crucial role in shaping the loss of symmetry of individuals even before symptoms of AD appear. Additionally, significant differences in CAI between AMC and SMC were observed, further indicating that genetic factors may influence early brain asymmetries in this at-risk group. Even atypical presentations are not frequent in ADAD; another possible explanation is that atypical AD forms, non-memory-dominant clinical presentation, are more predominant in the absence of the *APOE* ε4 allele, according to some researcher findings,^52-55^ which could also explain the higher brain asymmetries in *APOE3/3* individuals. However, other studies have shown similar frequencies between typical and atypical AD.^56-58^ Finally, Whitwell *et al*.^59^ conclude that typical AD showed higher *APOE* ε4 frequencies than atypical AD only between a range of ages. Thus, there is no clear statement in the scientific community. These overall findings in *APOE* genotype and brain asymmetry underscore the importance of considering genetic factors in understanding AD pathogenesis and brain asymmetry.

The increase in NfL levels is not specific to Alzheimer’s disease but serves as a strong indicator of neurodegeneration,^60^ with research studies associating elevated NfL with disease progression in AD. Also, elevated NfL levels in AMC may suggest that the individual is closer to transitioning to SMC.^18,26,61,62^ These findings are also associated with cortical atrophy.^63,64^ Although NfL has been proposed as a prognostic marker, it has yet to be validated and is not yet established in clinical practice. In contrast, neuroimaging markers, such as CAI, offer the advantage of providing valuable regional information, unlike biochemical biomarkers, such as NfL. The correlation between CAI and NfL levels in both cohorts suggests that CAI reflects the advanced brain neurodegeneration due to AD and can track the underlying neurodegenerative process. Furthermore, NfL levels and MMSE scores have been reported to correlate with cortical thinning due to atrophy caused by AD progression.^18,26,30,65,66^ In line with these findings, our work shows that brain asymmetry follows these patterns. This suggests that not only global brain atrophy is related to biochemical biomarkers that indicate neurodegeneration processes such as NfL level and cognitive changes due to dementia such as MMSE, but also brain asymmetry. The association between brain asymmetry and EYO, where brain asymmetry increases as individuals approach the expected age of symptom onset, is another evidence of CAI’s potential as a clinically useful biomarker for studying the disease progression across all stages of AD. Finally, our work also demonstrates that CAI increases over time, suggesting that AD progression leads to a loss of brain symmetry. SMC-AD individuals presented the most increase in brain asymmetry over time. Meanwhile, CTR presented almost a flat trajectory. Previous studies have suggested that the cerebral cortex thins asymmetrically throughout life, including during healthy aging, with an accelerated thinning in the presence of AD.^4,67^ These findings align with the results obtained in this ADAD study. Altogether, these results highlight the CAI as a potential biomarker for tracking disease progression along the disease continuum.

Our study has some limitations. Demographic differences, mainly in EYO, may impact the reproducibility of results across cohorts. The latest stages of the disease are underrepresented in this study. In the Clinic Barcelona cohort, we do not have participants *with PSEN2*, and in the DIAN-OBS cohort, we have a reduced number of individuals with PSEN2; therefore, we were unable to study CAI in *PSEN2* in detail. In this study, we only include ADAD individuals and do not compare the CAI results between ADAD and sporadic AD.

In conclusion, this study provides evidence that brain asymmetry is a potential biomarker for early ADAD detection and monitoring, tracking disease-related neuroanatomical changes. CAI correlates with other biomarkers of neurodegeneration, such as NfL, and cognition, such as MMSE. The influence of the *APOE* genotype on CAI further underscores the genetic factors that contribute to brain asymmetry.

## Supplementary Material

fcaf488_Supplementary_Data

## Appendix

### List of The Dominantly Inherited Alzheimer Network (DIAN) authors:

Randall Bateman, Alisha J. Daniels, Laura Courtney, Angela Ziegemeier, Karina Skrbec, Cortaiga Hellm, Mariana Martin, Ellen Ziegemeier, Jamie Bartzel, Eric McDade, Jorge J. Llibre-Guerra, Charlene Supnet-Bell, Chengie Xiong, Xiong Xu, Ruijin Lu, Guoqiao Wang, Yan Li, Yuzheng Nie, Emily Gremminger, Richard J. Perrin, Erin Franklin, Laura Ibanez, Gina Jerome, Jennifer Stauber, Bryce Baker, Matthew Minton, Carlos Cruchaga, Alison M. Goate, Alan E. Renton, Danielle M. Picarello, Brian Fulton-Howard, Tammie L.S. Benzinger, Brian A. Gordon, Jessica Banks, Russ Hornbeck, Allison Chen, Charles Chen, Shaney Flores, Manu Goyal, Diana Hobbs, Nelly Joseph-Mathurin, Kelley Jackson, Sarah Keefe, Deborah Koudelis, Parinaz Massoumzadeh, Austin McCullough, Nicole McKay, Joyce Nicklaus, Christine Pulizos, Qing Wang, Edita Sabaredzovic, Jalen Scott, Ashlee Simmons, Jacqueline Rizzo, Andrei Vlassenko, Yong Wang, Jason Hassenstab, Jennifer Smith, Sarah Stout, Andrew J. Aschenbrenner, Celeste M. Karch, Jacob Marsh, John C. Morris, David M. Holtzman, Nicolas R. Barthélemy, Jinbin Xu, Sarah B. Berman, Snezana Ikonomovic, Gregory S. Day, Neill R. Graff-Radford, Martin Farlow, Jasmeer P. Chhatwal, Courtney Maa, Takeshi Ikeuchi, Kensaku Kasuga, Takanobu Ishiguro, Kenji Ishii, Michio Senda, Yoshiki Niimi, Edward D. Huey, Stephen Salloway, Emma Devenney, Peter R. Schofield, William S. Brooks, Jacob A. Bechara, Ralph Martins, Nick C. Fox, David M. Cash, Natalie S. Ryan, Mathias Jucker, Christoph Laske, Reda Timofejavaite, Elke Kuder-Buletta, Susanne Graber-Sultan, Christian la Fougère, Gerald Reischl, Ulrike Obermueller, Johannes Levin, Yvonne Rödenbeck, Jonathan Vöglein, Jae-Hong Lee, Jee Hoon Roh, Paolo Vitali, Ricardo F. Allegri, Patricio Chrem Mendez, Ezequiel Surace, Silvia Vazquez, David Aguillon, Yudy Milena Leon, Laura Ramirez, Laura Serna, Ana Baena, Yamile Bocanegra, Allan I. Levey, Erik C.B. Johnson, Nicholas T. Seyfried, John Ringman, Anne M. Fagan, Hiroshi Mori, Colin Masters, James M. Noble, Raquel Sanchez-Valle and Francisco Lopera.
