# Supplementary material for: Cortical asymmetry in autosomal dominant Alzheimer’s disease progression
Source: Brain Commun. 2025 Dec 19;8(1):fcaf488. doi: 10.1093/braincomms/fcaf488 (PMC12781868; doi:10.1093/braincomms/fcaf488)
Supplement: fcaf488_Supplementary_Data [file fcaf488_supplementary_data.docx]

# Supplementary Material

**Cortical asymmetry in autosomal dominant Alzheimer’s disease progression**

Agnès Pérez-Millan, Neus Falgàs, Beatriz Bosch, Sergi Borrego-Écija, Anna Antonell, Guadalupe Fernández‐Villullas, Diana Esteller-Gauxax, Adrià Tort-Merino, Núria Bargalló, Mircea Balasa_,_ Albert Lladó, David Aguillon, Patricio Chrem, Gregory S. Day, Emma Devenney, Edward Huey, Takeshi Ikeuchi, Mathias Jucker, Kensaku Kasuga, Jonathan Vöglein, Jee Hoon Roh, Paolo Vitali, Ana Luisa Sosa Ortiz, Jorge J. Llibre-Guerra, Brian A. Gordon, Eric McDade, Randall J. Bateman, Raquel Sánchez-Valle, The Dominantly Inherited Alzheimer Network (DIAN)

^*^ Corresponding authors:

Agnès Pérez-Millan, PhD

Alzheimer’s disease and other cognitive disorders unit. Hospital Clinic de Barcelona, Institut d’Investigacions Biomèdiques August Pi I Sunyer (IDIBAPS), Villarroel, 170 08036 Barcelona (Spain). Tel: +34 932275785

[agperez@recerca.clinic.cat](mailto:agperez@recerca.clinic.cat)

and

Raquel Sánchez-Valle, MD, PhD

Alzheimer’s disease and other cognitive disorders unit. Hospital Clinic de Barcelona, Institut d’Investigacions Biomèdiques August Pi I Sunyer (IDIBAPS), Villarroel, 170 08036 Barcelona (Spain). Tel: +34 932275785

[rsanchez@clinic.cat](mailto:rsanchez@clinic.cat)

**CAI by Clinical Status in DIAN-OBS Cohort**

**
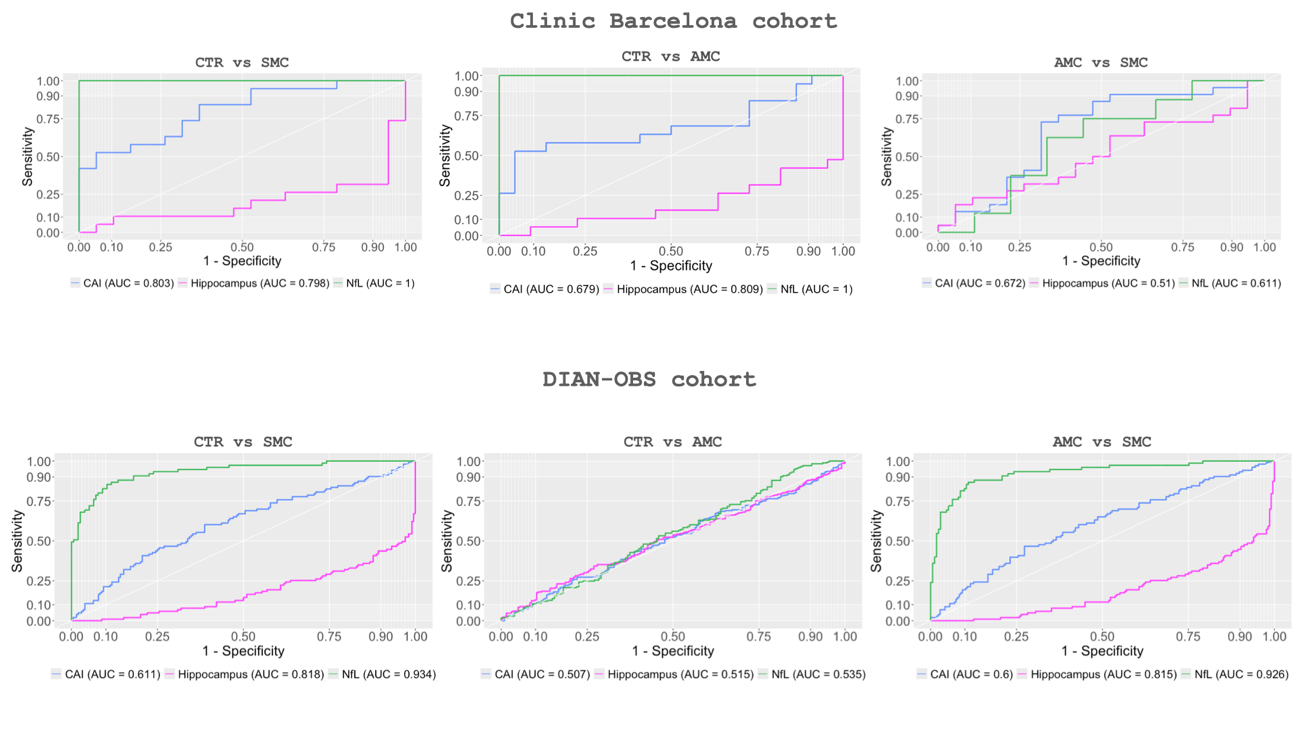
**

**Figure 1:** ROC curves A) Clinic Barcelona cohort B) DIAN-OBS cohort. Asymptomatic mutation carriers (AMC), symptomatic mutation carriers (SMC)and healthy controls (CTR)

**CAI by Clinical Status in DIAN-OBS Cohort**

**
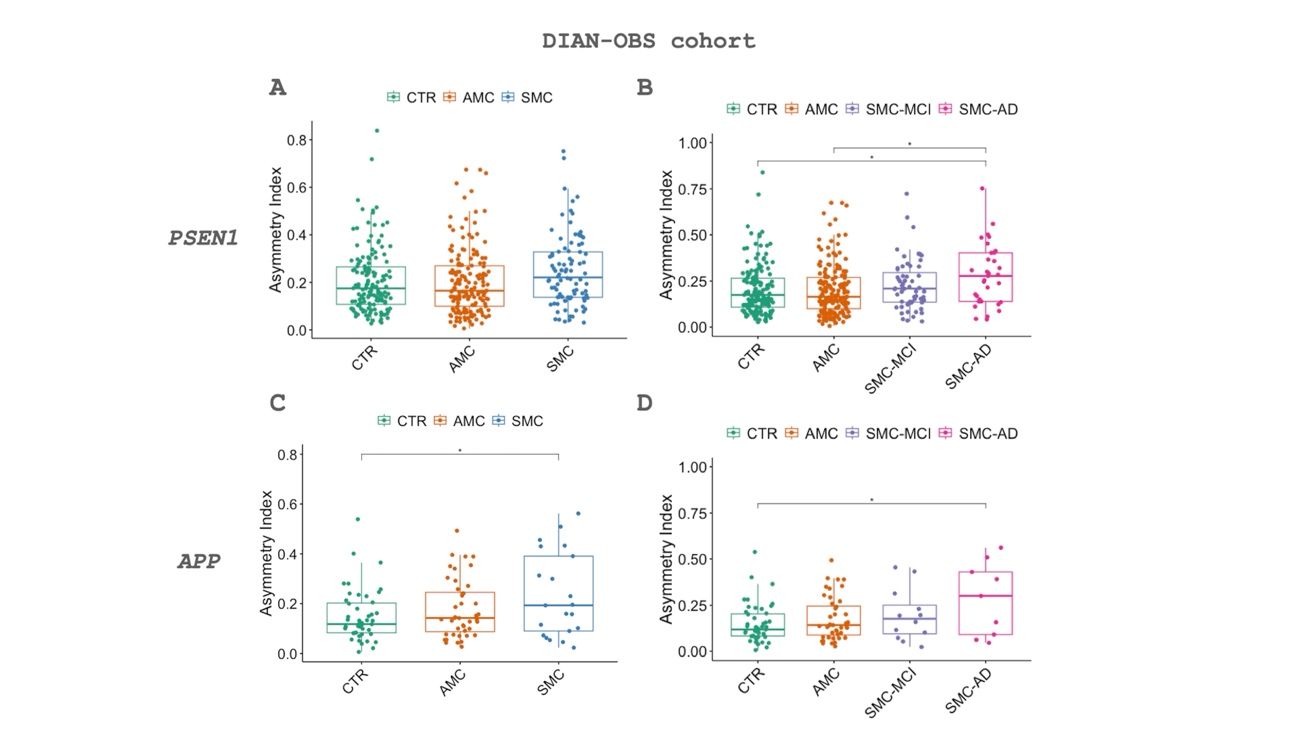
**

**Figure 2:** Asymmetric index showing significant differences between groups in DIAN-OBS cohort estimated with a permutation test. A) and B) Results for PSEN1 healthy controls (CTR) (N=146), asymptomatic mutation carriers (AMC) (N=166), symptomatic mutation carriers (SMC) (N=92), SMC-MCI (N=59) and SMC-AD (N=33) C) and D) Results for APP individuals CTR (N=47), AMC (N=44), SMC (N=21), SMC-MCI (N=12) and SMC-AD (N=9). Each data point represents the asymmetric index of one individual participant. Symbols indicate significance levels: * p < 0.05.

**CAI by *APOE* genotype in DIAN-OBS Cohort**

## *
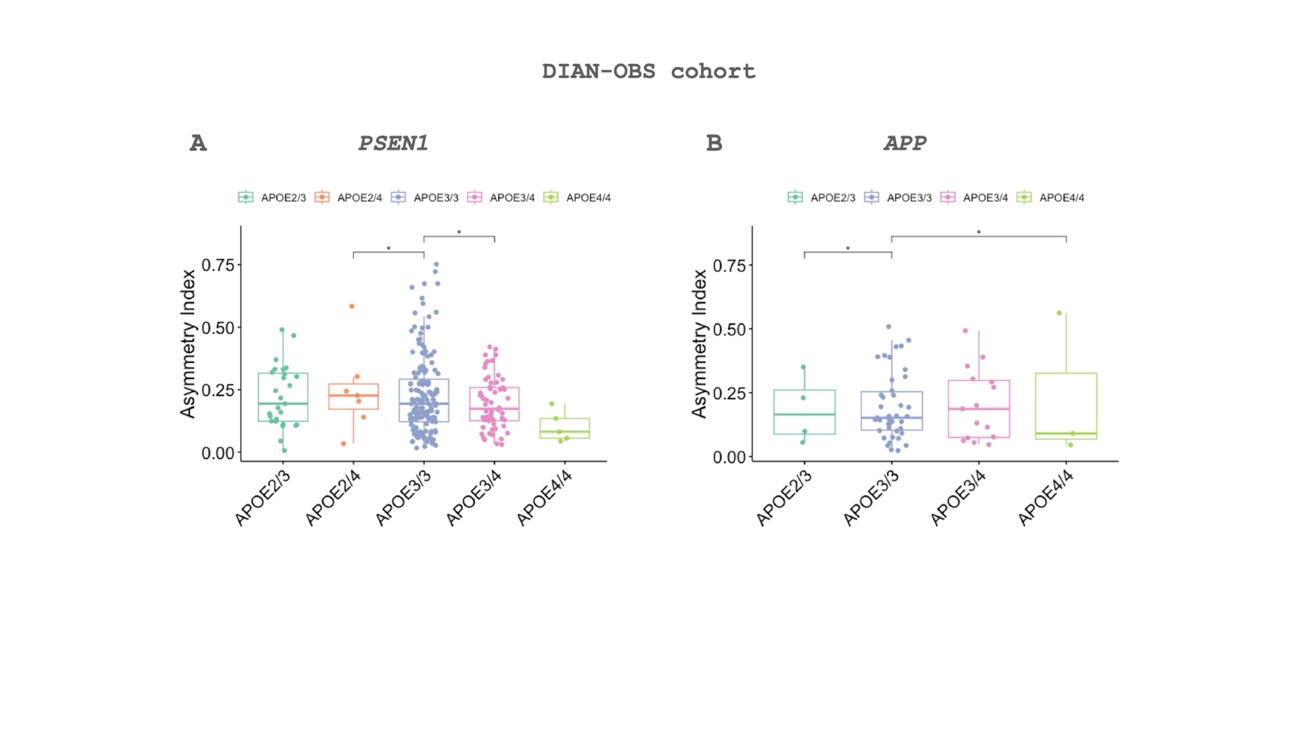
Figure 3: Asymmetric index showing significant differences between APOE genotypes in DIAN-OBS cohort analysed with a permutation test, differences between APOE genotypes for mutation carriers (asymptomatic mutation carriers (AMC) and symptomatic mutation carriers (SMC)). A) Results for PSEN1 individuals (N=256). B) Results for APP individuals (N=65). Each data point represents the asymmetric index of one individual participant. Symbols indicate significance levels: * p < 0.05.*
